# Improving Conformational Stability and Bacterial Membrane Interactions of Antimicrobial Peptides with Amphipathic Helical Structure

**DOI:** 10.21203/rs.3.rs-7340189/v1

**Published:** 2025-08-20

**Authors:** Ahmad Habibie, Rizki Amalia Putri, Respati Tri Swasono, Endah Retnaningrum, Prajnaparamita Dhar, Krzysztof Kuczera, Tri Joko Raharjo, Teruna Siahaan

**Affiliations:** Gadjah Mada University; Gadjah Mada University; Gadjah Mada University; Gadjah Mada University; The University of Kansas; The University of Kansas; Gadjah Mada University; The University of Kansas

**Keywords:** antimicrobial peptides, antimicrobial resistance, macroalgae, helical structure, membrane binding, CD, NMR, MD simulations

## Abstract

Antimicrobial resistance (AMR) has become a massive concern because it causes the loss of human life and an economic burden in many parts of the world. Antimicrobial peptides (AMPs) can be investigated as an alternative solution to combat AMR because their mechanism has the potential to reduce microbe resistance. In this study, the native P01 peptide from *Chondrus crispus* macroalgae was modified to P01.1, P01.2, and P01.3 peptides via residue mutations and capping of the N- and C-termini to systematically improve their a-helical content, bacterial membrane interaction, and antibacterial activity. C-terminus amidation and mutations to remove helix breaker residues in P01 to give P01.1 peptide enhanced its a-helical stability. Acetylation of the N-terminus P01.1 to give P01.2 peptide further enhanced the a-helical content of the peptide. Mutations of low-to-high helical former residues in P01.2 to give P01.3 peptide further improve its a-helical stability. The binding activity of peptides to a model of Gram-positive membrane is in the following order P01.3 > P01.2 > P01.1 > P01; this is correlated with their antibacterial activity against Gram-positive *S. aureus* with MICs in the following order P01.3 = 15.63 mg/mL > P01.2 = 125 mg/mL > P01.1 and P01 larger than 250 mg/mL. In a model of Gram-negative membrane, the peptide-membrane binding is in the following order P01.3 = P01.2 > P01.1 > P01; however, P01.3, P01.2, and P01.1 have the same antibacterial activity against Gram-negative *E.coli* (MIC = 3.91 mg/mL) while P01 has no activity. In conclusion, the a-helical stability and amphipathicity of the peptide have correlation with the membrane binding and antibacterial activity of the peptide.

## Introduction

Antimicrobial resistance (AMR) has now become a major concern in global health which causes millions of deaths annually. From the economic point of view, AMR was predicted to affect the loss of GDP up to 3.4 trillion USD by 2030 and it may increase to 3.9 trillion USD by 2050 [1]. Since the first antibiotic discovery in the 1950s, many antibiotics have been developed and widely administered to patients and animals. Unfortunately, excessive use of antibiotics has a significant impact on generating AMR [1]. Therefore, there is an urgent need to discover and develop new antimicrobial agents that overcome the drug resistance problem.

The mechanism of action of conventional antibiotics is mainly through the interference of immune modulation for the microbial species; however, microbes have mechanisms to induce antibiotic resistance [2]. Antimicrobial peptides (AMPs) are promising agents to kill microbes via non-receptor-mediated membrane damage, that reduces the resistance probability. AMPs exhibited a broad spectrum of activity against Gram-negative and Gram-positive bacteria, fungi, and even viruses. With these advantages, AMPs have great potential as new antimicrobial agents.

Natural AMPs function as one of the immune defense systems of animals against bacterial infections [3]. AMPs were isolated from various organism species with a variety of characteristics such as cationic in nature with amphipathic α-helix or β-sheet secondary structures [4]. Although AMPs exhibited activity against broad-spectrum bacteria, their natural structures have low activity and have not been optimized. The unoptimized AMPs are also enzymatically unstable and have low secondary structure stability. Therefore, there is need to improve their conformational and enzymatic stabilities for developing them as antibacterial agents to treat patients. To overcome the instability of AMPs, several peptides such as LL-37, magainin-2, and Aurein can be optimized using *de novo* and rational design methods to enhance structural and enzymatic stability as well as biological activity [5, 6]. In addition, N- and C-terminal modifications and amino acid mutations can also be implemented to improve the potency of AMPs [7]. Thus, the hypothesis is that improving the amphipathic α-helix structure of AMPs by mutation and capping strategies can improve the antimicrobial activity of AMPs.

Previously, we found that the P01 peptide from the *Chondrus crispus* hydrolysate protein has antibacterial activity [8]. The bioinformatics analysis indicated that the P01 peptide exhibited an α-helical structure with imperfect amphipathic property [8]. In this study, the P01 peptide structure was optimized via derivatization to enhance the amphipathicity and helical structure of P01 analogs by N- and/or C-termini capping and amino acid mutations to helix-forming amino acids. In this work, the secondary structural and dimerization properties of P01 peptide and three of its derivatives (i.e., P01.1, P01.2, and P01.3) were determined by circular dichroism (CD), two-dimensional Nuclear Magnetic Resonance (2D NMR), and molecular dynamics simulations. These biophysical properties were corelated with studies to determine the biological activities of these peptides to inhibit the growth of *Escherichia coli* (*E. coli*) and *Staphylococcus aureus* (*S. aureus*) bacteria. Finally, the efficiency of these peptides to limit bacterial growth was corelated with the membrane-binding properties of these peptides, which were evaluated using model membranes formed on a Langmuir trough.

## Results and Discussion

### Design of AMP Derivatives from Macroalgae Chondrus Crispus

**Figure 1:**
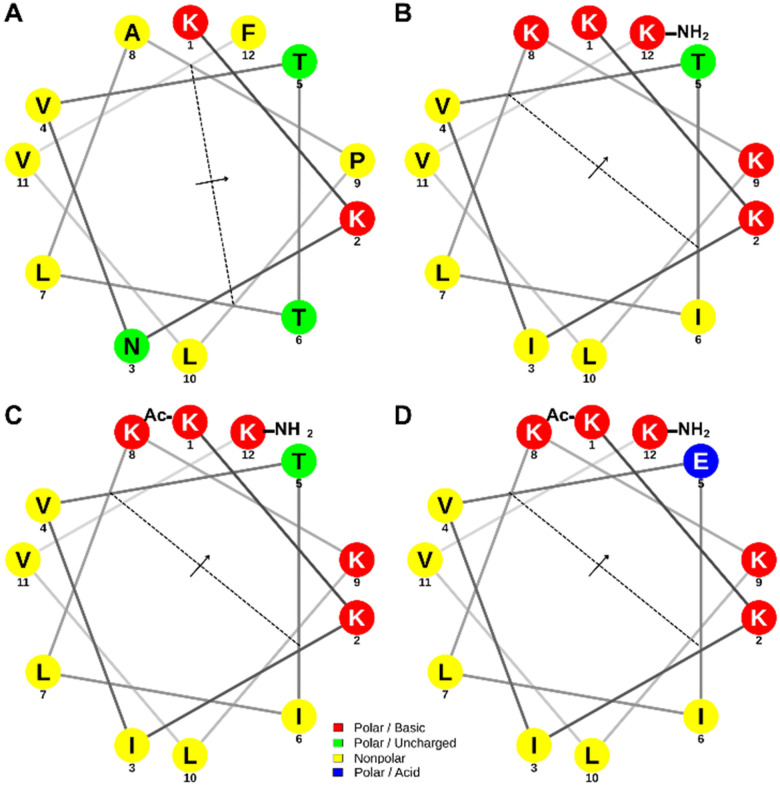
The helical wheel and net projection of (A) P01, (B) P01.1, (C) P01.2, (D) P01.3 peptides. The arrow is directed to the peptide hydrophilic face and the dashed line divides the hydrophobic and hydrophilic faces. The polar basic residues with a positive charge and polar acidic residues with a negative charge are indicated as red and blue colors, respectively. The polar uncharged and non-polar residues are marked as green and yellow colors, respectively. The helical diagram was generated using NetWheels: Peptides Helical Wheel and Net projections maker (http://www.lbqp.unb.br/NetWheels/).

P01 peptide was discovered using proteomic approach from proteins of macroalgae *Chondrus crispus* as the parent peptide [8]. As shown in Fig. 1A, P01 peptide showed a lack of amphipathic structure with two hydrophobic residues (Pro9 and Phe12) in hydrophobic face and one hydrophilic residue (Asn3) in hydrophilic face of the peptide. As seen in Table 1, the parent P01 peptide has + 2 total charges with two lysine residues and a hydrophobicity value (< H>) of 0.550. and a low mean hydrophobic moment (<μH>) of 0.246. A large value of μH means that the helical structure is amphipathic perpendicular to its axis. The peptide derivatives were obtained by sequential modification to obtain better properties. As shown in Table 1, the parent and derivative peptides were predicted to be non-toxic peptides.

P01.1 peptide was derived from P01 peptide with several mutations in the hydrophilic and hydrophobic faces to obtain an amphipathic helical structure. Mutation of Asn3 and Thr6 with Ile3 and Ile6, respectively, was intended to increase amphipathicity of P01.1 peptide (Fig. 1B). In the hydrophilic face of P01 peptide, Ala8 and Pro9 were mutated with Lys8 and Lys9, respectively, to increase the total charge and amphipathicity in P01.1 peptide. The C-terminus of P01.1 peptide was capped with amide group to stabilize peptide helix structure [9]. As shown in Table 1, P01.1 peptide has a lower hydrophobicity and higher total charge of 0.396 and + 6, respectively, as an impact of lysine substitutions; this peptide exhibited a good amphipathic structure with five lysine and one threonine residues at the hydrophilic face and several residues (i.e., Val, Leu, Ile) at the hydrophobic face. The P01.1 peptide has a higher μH value (0.746) than that of P01 peptide (0.246) (Table 1).

The P01.2 peptide has a similar sequence as P01.1 but it was capped at both N- and C-termini with acetyl and amide groups, respectively, to increase the helicity and conformational stability of the peptide (Fig. 1C) [9]. P01.2 has a lower total charge of + 5 than that of P01.1 peptide (+ 6) as the impact of N-terminal capping. The P01.2 peptide exhibited similar H and μH values of 0.396 and 0.746, respectively, with P01.1 peptide (Table 1).

P01.3 peptide was modified by substituting Thr5 with Glu5 in the P01.2 peptide in which the Glu5 residue is a higher helix inducer than the Thr5 residue (Fig. 1D). This produced the enhancement of amphipathic property in P01.3 peptide with H and μH values of 0.321 and 0.821, respectively (Table 1). The substitution of the Thr5 residue in P01.2 peptide to Glu5 in P01.3 peptide decrease the charge from + 5 to + 4.

### Secondary Structure Analysis of Peptides using CD

The peptide secondary structures were determined by circular dichroism (CD) spectrophotometry. The α-helical structure of peptide is expected to exhibit three characteristic signals with a maximum at 190 nm, and two minima at 222 and 208 nm that correspond to the n−π* and π−π* electronic transitions of the α-helical structure. TFE was used to mimic the membrane environment for inducing the helical structure. The helical structure formation was observed by varying TFE concentrations from 0–50%. The fractional helicity (*f*_*H*_) was determined to monitor the helical structure formation of peptide derivatives in every TFE concentration in 10 mM sodium phosphate buffer.

**Figure 2:**
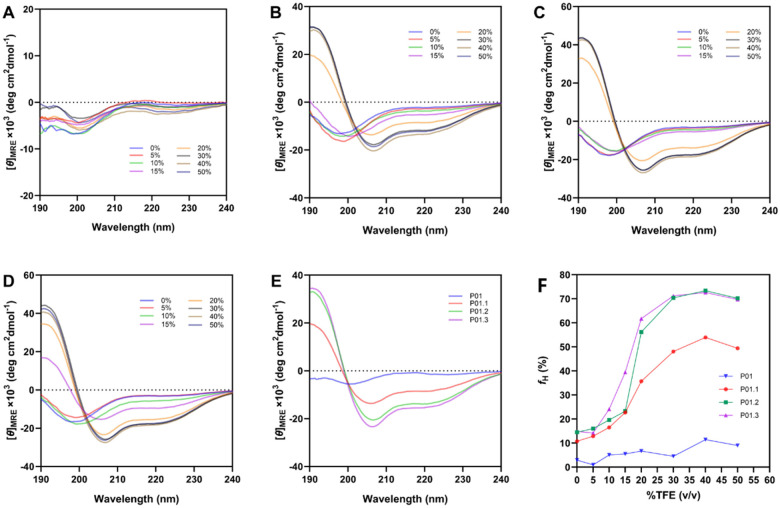
Circular dichroism spectra of (A) P01, (B) P01.1, (C) P01.2, and (D) P01.3 in 0–50% TFE in water (v/v). (E) The comparison of CD spectra of all peptides in 20% TFE in water (v/v). (F) The mean helicity of all peptides upon increasing concentrations of TFE.

CD spectra of P01 peptide exhibited mostly a random coil nature with a minimum absorption around 200 nm without TFE (Fig. 2A). The spectral minimum at 200 nm decreased while the spectral minimum at 222 nm increased when the TFE concentrations were increased from 10–50%; however, the classical critical minima at 208 and 222 nm for helical structure were not observed. The low helical content of P01 peptide may be due to the presence of the Pro9 residue as a helical breaker residue. P01.1 peptide mostly exhibited a random coiled spectra at 0–15% TFE with increasing the signal at 222 nm followed by decreasing signal at 200 nm as the TFE amounts increased (Fig. 2B). A clear CD helical spectral characteristic with minima at 208 and 222 nm were observed at 20% TFE; these helical minima were significantly more intense as the TFE concentration reached 50%. For P01.2 peptide, the signal for a helical pattern was not observed in 0–5% TFE (Fig. 2C); however, the signal minimum at 222 nm was intensified followed by a decreased intensity at 200 nm at 10–15% TFE concentrations. The helical pattern with minima at 208 and 222 nm was clearly observed at 20% TFE for P01.2 peptide; thus, a clear transition between random coil and helical structures was between 15% and 20% TFE. P01.3 peptide showed mostly random coil signal at 0% TFE (Fig. 2D) and the increase in TFE concentrations to 5–10% intensified the signal at 222 nm. The characteristic helical pattern with minima at 208 and 222 nm was observed at 15% of TFE, where the transition between a mostly random coil structure to mostly helical structure was between 10–15% TFE. The helix signal pattern of P01.3 peptide was maximized at 20% TFE.

To summarize the impact of chemical modification on the peptide helicity, the intensities of the helical signal of all peptides were compared at 20% TFE (Fig. 2E). P01 peptide (blue curve) mostly has a spectrum characteristic of a random coil, with a small fraction of helicity. Moreover, the presence of the Pro9 residue as a helical breaker in P01 peptide contributed to its low propensity to form a helical structure.

The first derivative, P01.1 peptide, obtained by substituting the Asn3, Thr6, and Pro9 residues in the P01 peptide with the Ile3, Ile6, and Lys9 residues, respectively, and capping the C-terminal with the amide group, showed a pronounced helical spectrum [9]. P01.2 peptide exhibited a higher signal intensity for a helical structure in 20% TFE than P01 and P01.1 peptides. The significant increase in helicity was also due to acetylation of the N-terminus. The final derivative, P01.3 peptide, exhibited the highest helix signal pattern because of the mutation of Thr5 to Glu5 residue. The potential salt bridge(s) from the Glu5 residue (*i*) to the Lys8 (*i + 3*) or Lys9 (*i + 4*) residue in the hydrophilic face of P01.3 peptide could stabilize the helical structure. A previous study showed that a salt bridge interaction at the (*i, i* + 3) or (*i, i* + 4) position could stabilize the helical structure; however, the salt bridge at (*i, i + 1*) or (*i, i + 2*) could destabilize the helix structure [10].

The effects of TFE concentrations on the helical content of each peptide were determined using the mean helicity (*f*_H_; Fig. 2F). In pure buffer, *f*_*H*_ values were in the following ranking order: P01 with *f*_*H*_ = 2.9% < P01.1 with *f*_*H*_ = 10.8% < P01.2 with *f*_*H*_ = 14.5% < P01.3 with *f*_*H*_ = 14.8% (Fig. 2F). The helicity of P01 peptide increased slightly to *f*_*H*_ of 4.5–6.7% when the TFE was increased to 10–30%; finally, the helicity content was maximized *f*_*H*_ of 9.0–11.4% at 40–50% TFE concentrations (Fig. 2F). P01.1 peptide has *f*_*H*_ values of 10.8–22.7% at 0–15% TFE, increasing nonlinearly to a value of 35.7% at 20% TFE followed by reaching maximum value of 48.1–53.9% at 30–50% TFE. Next, P01.2 peptide has *f*_*H*_ = 19.6–23.3% in 10–15% TFE while the helical content dramatically increased to 56.1% in 20% TFE followed by a further increase, reaching maximum *f*_*H*_ values of 70.2–70.4% in 40–50% TFE. Finally, P01.3 peptide has higher helicity (*f*_*H*_ = 39.4%) than P01.2 peptide (*f*_*H*_ = 24.1%) at 15% TFE concentration (Fig. 2F), while P01.2 peptide has almost a similar helicity value with P01.3 peptide in 30–50% TFE.

The correlation between the helicity content as a function of TFE concentration has not been well understood and it predominantly also depends on peptide sequences. TFE induces helicity by decreasing interaction between peptide amides and water [11]. Water destabilizes the helix conformation by intermolecular hydrogen bond interactions with peptide amide bonds to break amide-carboxyl intramolecular hydrogen bonds in the helix backbone [12]. Previous studies suggested that the increase in helical properties was due to increase of TFE concentrations to strengthen intramolecular hydrogen bonds [11].

### The Effect of Temperature on Peptide Physical Stability

**Figure 3:**
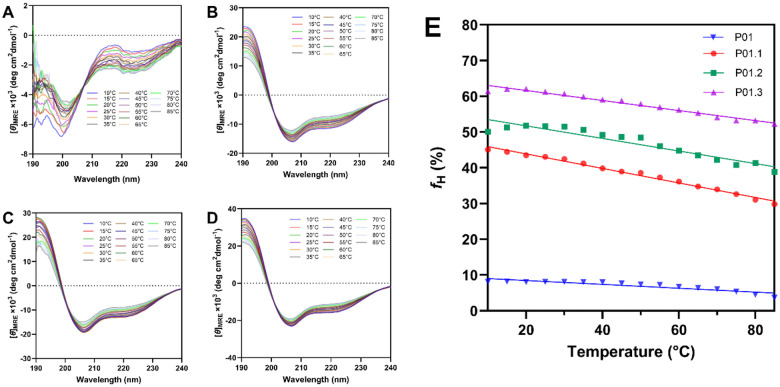
The CD spectra of each peptide during temperature-dependent studies from 10–85 °C for (A) P01, (B) P01.1 (C) P01.2, (D) P01.3. (E) The mean helicity of peptide derivatives as a function of temperature. The helicity was reduced as a function of temperature.

The physical stability evaluations of each peptide were monitored using CD spectrophotometry at different temperatures and pHs. In this case, the stability studies were conducted at 20% TFE in 10 mM sodium phosphate buffer (v/v). The temperature-dependent study was performed at 10–85 °C at pH 7.0. Although P01 peptide showed mostly a random coil structure at 10 °C, the increase in temperature dramatically increased the intensity minimum at 222 nm followed by the decrease in intensity of 200 nm (Fig. 3A), suggesting there was structural change in P01 peptide upon the increase in temperature. For P01.1 peptide, the increase in temperature from 10 to 85 °C decreased the minima at 208 and 222 nm while the maximum at 190 nm were increased (Fig. 3B). A similar trend was observed for P01.2 peptide (Fig. 3C) and P01.3 peptide (Fig. 3D) with the change in temperature from 10 to 85 °C.

To quantify the structural change as a function of temperature, the mean helicity values (*f*_*H*_) were plotted against temperatures for each peptide (Fig. 3E). At the initial temperature at 10 °C, P01 peptide has helicity with *f*_*H*_ of 9.1%, and as the temperature was increased, the helical content decreased to *f*_*H*_ of 3.7% at 85°C (Fig. 3E). Thus, the decrease in helicity per degree of temperature (D*f*_*H*_/DT) was 0.07%/deg. This change was small because P01 peptide already has a low helical structure at the low temperature.

The stabilities of helical structure of P01.1, P01.2, and P01.3 peptides were compared as a function of temperature (Fig. 3E). At 10 °C, the rank of helicity of P01.3 > P01.2 > P01.1 with *f*_*H*_ of 61.4, 50.0, and 45.1%, respectively. To compare the helical stability of these three peptides as a function of temperature, the helicity lost between 20–85 °C and D*f*_*H*_/DT were determined. The results showed that P01.3 peptide has the lowest helicity loss of 9.8% with D*f*_*H*_/DT = 0.15%/deg. Next, P01.2 peptide has the helicity loss of 12.9% and D*f*_*H*_/DT = 0.20%/deg. Finally, P01.1 peptide has helicity loss of 13.7% *f*_*H*_ and D*f*_*H*_/DT = 0.21%/deg. Overall, these studies showed that P01.3 has the highest thermal stability followed by P01.2 and P01.1 peptides. Thus, modification in the P01 peptide was successful in improving the helical stability of its derivatives.

### The Effect of pH on Peptide Physical Stability

**Figure 4:**
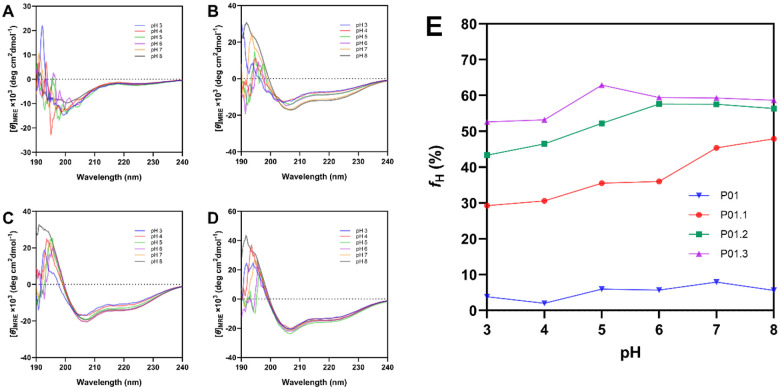
pH-dependent studies of (A) P01, (B) P01.1, (C) P01.2, (D) P01.3 peptides from pH 3.0 to 8.0. (E) The mean helicity of analog peptides in pH 3.0 to 8.0.

The pH-dependent stability assay was also performed at pH 3.0 to 8.0. A 100 mM phosphate-citrate buffer was used to adjust the pH of the peptide solution. As shown in Fig. 4A, because the P01 peptide exhibited a random coil structure, no significant signal pattern change was observed in the peptide signals during the test at pH 3–8. In Fig. 4E, P01 exhibited *f*_*H*_ of 5.62% at pH 8.0 and slightly increased at pH 7 to *f*_*H*_ of 7.94%. At more acidic conditions, P01 fractional helicity fluctuated from 5.70% at pH 6.0 to 5.99% at pH 5.0. The fractional helicity decreased to 3.84 and 2.00% at pH 3.0 and 2.0, respectively.

As shown in Fig. 4B, the P01.1 peptide exhibited good stability at pHs 7.0 and 8.0 with fractional helicity of 47.92 and 45.40, respectively. The fractional helicity significantly decreased at 6.0 and 5.0 to 36.02 and 35.52%. The fractional helicity significantly decreased further at pHs 4.0 and 3.0 to 30.59% and 29.28%, respectively.

P01.2 peptide exhibited a more stable helical structure than P01.1 with relatively similar signal intensity at pH 6.0–8.0 with fractional helicities of 56.37–57.60% (Fig. 4C). The signal gradually decreased at more acidic conditions (pH 3.0–5.0) with *f*_*H*_ of 52.25% at pH 5.0, 46.49% at pH4.0, and 43.34% at pH 3.0. The *f*_*H*_ decreased in P01.1 and P01.2 are possibly due to electrostatic repulsion between lysine residue at their fully charged states.

P01.3 peptide exhibited the most stable helix structure than other derivative peptides with small signal intensity change (Fig. 4D). At pH 8.0–6.0, P01.3 peptide exhibited very similar signal intensity with *f*_*H*_ around 58.71–59.46%. The signal intensity slightly increased at pH 5.0 with *f*_*H*_ of 62.92% followed by a decrease at pH 4.0 and 3.0 to *f*_*H*_ of 53.23 and 52.63%, respectively. Glutamic acid can stabilize and increase the helicity by intramolecular interactions with lysine in peptide hydrophilic face [13]. At acidic conditions, glutamic acid residue exhibited a neutral charge, especially at pH below its pKa (~ 4.25), causing P01.3 peptide to lower its helicity [14].

### Dimerization Studies of Peptides

**Figure 5:**
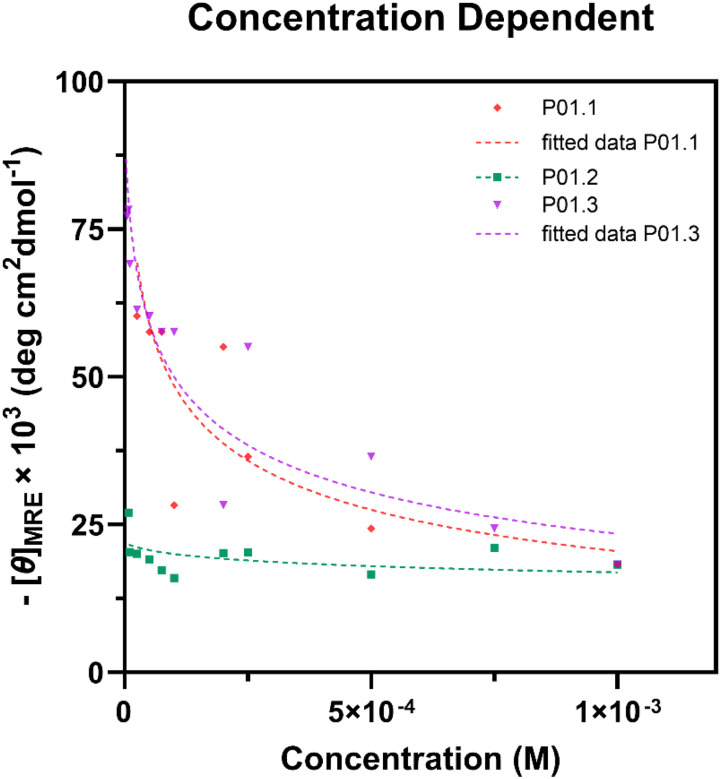
Concentration-dependent CD spectrophotometry of derivative peptides with a linear least-square fitting method using equation 3.

Amphipathic helical peptides have the potential to form dimers and oligomers in aqueous solution. In several cases, the hydrophobic face of peptides generally interacts to form a hydrophobic core of oligomer [15]. Here, we studied the potential dimerization of each peptide using CD spectroscopy in a concentration-dependent manner at 222 nm. The dissociation constant (K_D_) was obtained by fitting Eq. 3 to observed data with the nonlinear least-square fitting method (Fig. 5) [16].

The increase in peptide concentrations of P01.1 and P01.3 peptides showed signal intensity shifts at 222 nm, which were due to the peptide dimer or oligomer formation (Fig. 5) [17]. The previous study showed that helical peptides possibly lose their helix structure in oligomer formation in high concentrations [18]. In contrast, P01.2 peptide exhibited a lack of concentration-dependent spectral change, suggesting that there was no dimerization or oligomerization present [19]. The results indicated that P01.2 was in a monomeric state in a concentration range of 7.5×10^− 4^ − 1×10^− 3^ M. According to these results, the K_D_ determination was limited to P01.1 and P01.3 dimers in the current report.

P01.3 peptide showed a dimer formation with K_D_ and COD of 5.15×10^− 5^ M and 0.99, respectively (Table 2). The stable dimer formation could be due to the formation of an intermolecular salt bridge between the Glu5 and Lysine residues for a dimer self-association [20]. In contrast, P01.1 peptide showed a high K_D_ of 7.69 ×10^− 4^ M with a COD of 0.92, indicating a lower probability to form a dimer. This is presumably due to a repulsive electrostatic interaction between positive charge residues in the hydrophilic face of the amphipathic helix [21]. Alternatively, P01.1 coefficient of could form a higher oligomer than a dimer. These results suggest that there is a correlation between amphipathicity and helicity of a peptide to its dimer or oligomer stability.

### Conformational Study of P01.3 Peptide by NMR and Molecular Dynamics Simulations

**Figure 6:**
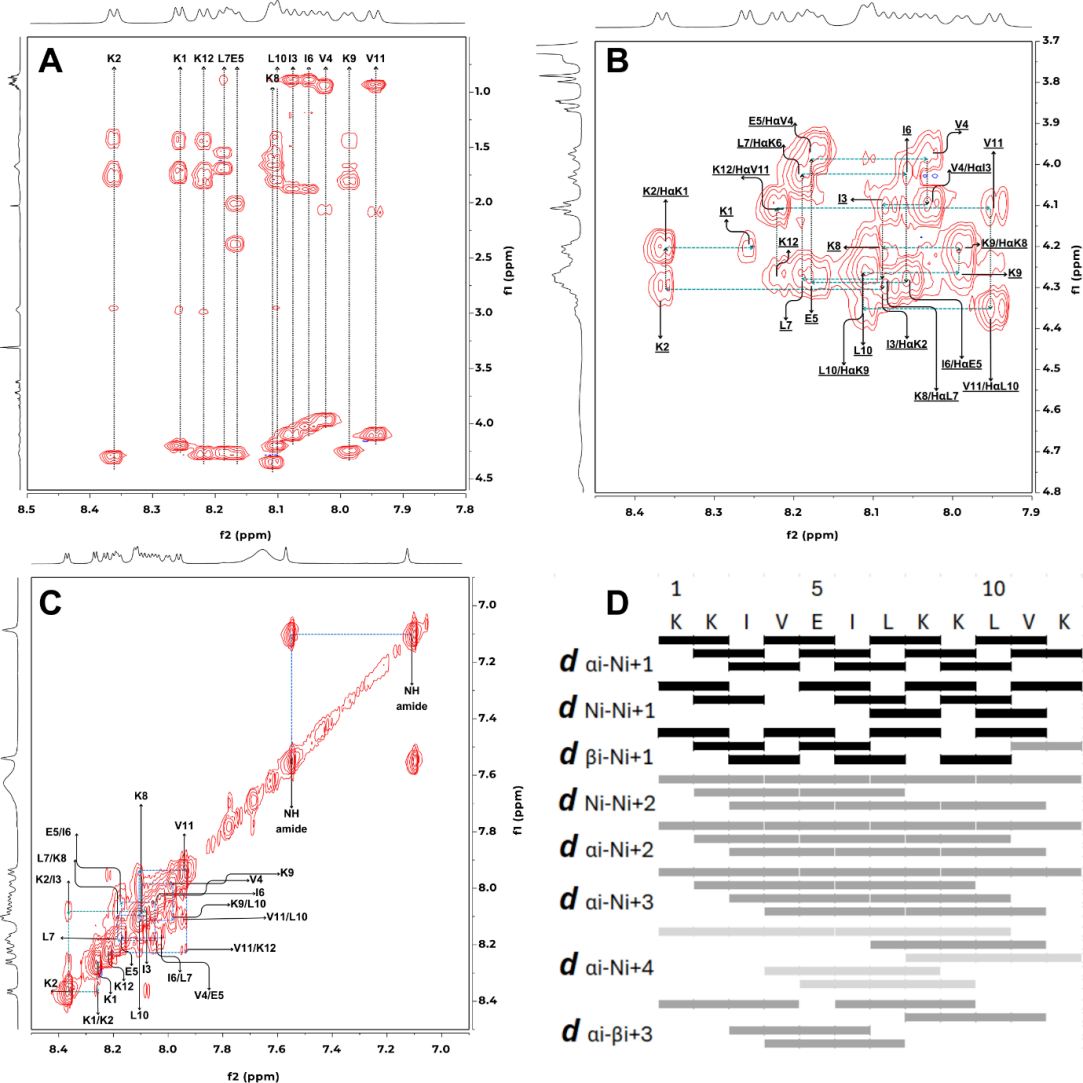
2D NMR spectrum assignment for P01.3 peptide: (A) amino acid assignment using TOCSY cross peak, (B) NH_(i)_-Hα(_i-1)_ assignment using NOESY cross peak, (C) NH-NH assignment using NOESY cross peak, and (D) intramolecular atom interaction strength of P01.3 peptide: strong interaction (black), medium interaction (grey), and weak interaction (light grey).

The 3D structure of the P01.3 peptide was determined by 2D-NMR (Fig. 6) and MD simulations, as it has the most stable and has highest helical content. 2D COSY and TOCSY (Fig. 6A) experiments were conducted to determine the proton assignments in each amino. The sequential assignment of each amino acid was done using the through space connectivities of NH-HCα (Fig. 6B) and HN-NH (Fig. 6C) for neighboring residues in the NOESY spectra. The assignments of all protons were shown on Table 3. Using TOCSY and NOESY spectra, the NH chemical shifts of several Lys residues were found at 8.37 (K_2_), 8.26 (K_1_), 8.22 (K_12_), 8.10 (K_8_), and 7.99 (K_9_) ppm. The lysine residue typically exhibited TOCSY connectivity from the NH proton to HCα, HCβ, HCγ, and HCδ protons (Fig. 6A). The NHs of Ile residues were observed at 8.06 (I_6_) and 8.08 (I_3_) ppm and these NHs have connectivities with HCα, HCβ, HCγ, and HCδ protons. The NH chemical shifts of leucine residues were observed at 8.19 (L_7_) and 8.11 (L_10_) ppm and the leucine residue has a similar connectivity pattern to isoleucine. Two valine residues showed their NHs at 7.94 (V_11_) and 8.03 (V_4_) ppm and have through bond connectivities from NH to HCα, HCβ, and HCγ protons. The NH of Glu5 (E_5_) was at 8.17 ppm and it exhibited connectivities to HCα, HCβ, and HCγ protons.

The assignment of amino acid sequence in P01.3 sequence was confirmed by the NOESY through-space connectivities of the HCα_(i)_−NH_(i+1)_-protons. A cross-peak between the HCα of Lys1 and the NH of Lys2 was observed and the HCα of Lys2 residue showed a cross-peak to the NH of Ile3 residue (Fig. 6B). A through-space connectivity from the HCα of Ile3 residue and the NH of Val4 residue was observed. The HCα of Val4 residue and the NH of Glu5 showed an NOE cross peak followed by a cross-peak between the HCα of Glu5 and the NH of Ile6 residues. The HCα_(i)_−NH_(i+1)_ connectivities were observed between (a) the HCα of Ile6 and the NH of Leu7; (b) the HCα of Leu7 and the NH of Lys8; (c) the HCα of Lys8 and the NH of Lys9; (d) the HCα of Lys9 and the NH of Leu10; (e) the HCα of Leu10 and the NH of Val11; and (f) the HCα of Val11 and the NH of Lys12.

The α-helical conformation of P01.3 peptide was determined using the through-space proton-proton interactions with signature crosspeaks such NH_(i)_−NH_(i+1)_, NH_(i)_−NH_(i+2)_, HCα_(i)_−NH_(i+2)_, HCα_(i)_−NH_(i+3)_, HCα_(i)_−NH_(i+4)_, and HCα_(i)_−HCβ_(i+3)_ (Figs. 6C–D). In Fig. 6C, the NH_(i)_−NH_(i+1)_ crosspeaks were observed between the following residues: (a) Lys1–Lys2, (b) Lys2–Ile3, (c) Val4–Glu5, (d) Glu5–Ile6, (e) Ile6–Leu7, (f) Leu7–Lys8, (g) Lys8–Lys9, (h) Lys9–Leu10, (i) Leu10–Val11, and (j) Val11–Lys12. Other connectivies were also observed for NH_(i)_−NH_(i+2)_, HCα_(i)_−NH_(i+2)_, HCα_(i)_−NH_(i+3)_, HCα_(i)_−NH_(i+4)_, and HCα_(i)_−HCβ_(i+3)_ (Fig. 6D). A potential helical break was observed between Ile3 and Val4 residues because there was no crosspeak between the NH of Ile3 and the NH of Val4. The ^3^J_NH(i)-Hα(i+1)_ coupling constants of the peptide were around 6.68 to 8.63 Hz, which were higher than 6.0 Hz for typical coupling constants for a helical structure. This higher coupling constants could be due to dynamic nature of the peptide in solution using NMR analysis with a deviation be around 0.7 Hz [22].

Integrations of the NOE signals were utilized to determine the intramolecular proton distances within the peptide. The volume of NOE crosspeak from two geminal protons was used as reference for a distance (d) of 1.7Å in Eq. (4). The result was correlated directly with NOE signal volume and classified into three categories, d < 2.7 Å (strong interaction), 2.7–3.5 (medium interaction), and 3.5–5.0 Å (weak interaction). The results showed that the peptide has an α-helix structure. The interproton distances were used in NMR-restrained MD simulations to determine the solution conformation of P01.3 peptide.

**Figure 7:**
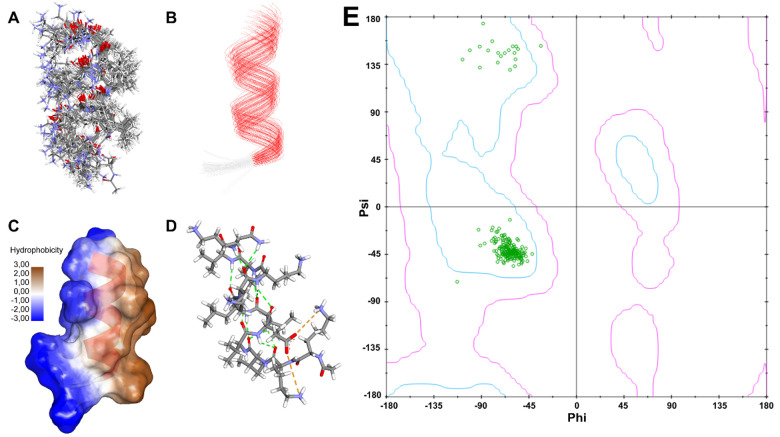
NMR refinement structures of P01.3 peptide generated using NMR restraint molecular dynamic simulations. The 20 lowest energy structures in (A) 3D structure and (B) wire secondary structures. The red wire showed the α-helix region, and the white wire showed a potential turn region. (C) The amphipathic structure of P01.3 peptide with hydrophilic and hydrophobic faces. (D) Intramolecular H-bond interactions network (green dash line) and salt bridge interaction (orange dash line) in P01.3 peptide with a helix conformation. All models have the N-terminal at the bottom to the C-terminal at the top. (E) Ramachandran plot to show the Phi and Psi relationship in the 20 lowest energy generated structures that indicate a helical Phi-Psi dihedral angles.

The 3D structure calculation was conducted by ARIAweb using NOE interproton distances from 12 from intra-residue, 21 sequential residues, 25 medium interactions, and 1 long-range interaci; in addition, 12 ^3^J_NH-Hα_ coupling constants were also used as constrains [23]. The best structure from MD simulations were analyzed by PROCHECK. The 20 lowest energy structures from the MD simulation have RMSDs of 0.69 ± 0.20 and 1.88 ± 0.31 for the backbone and all heavy atoms, respectively. The generated structures (Figs. 7A–B) have minimal distance violation from the NMR restraints with the helical conformation concentrated at the C-terminal region (Top region, Fig. 7B). The final structure has a hydrogen bonding network along the backbone between *i* and *i + 4* residues as one of the characteristics for a well-defined α-helix. The amphiphatic nature of the helix was observed in Fig. 7C. Additionally, a salt bridge interaction was observed between the side chain of Lys1 and the side chain of Glu5 residues or between the side chain of Lys3 with the side chain of Glu5 residues (Fig. 7D). Ramachandran plot (Fig. 7E) was generated to display the Phi and Psi angles congregated at the helical region. Although there was a potential helical break at Ile3-Val4 from the NMR data, the MD simulations result did not show any helical break in the resulted NMR structure of P01.3 peptide (Fig. 7C).

### Membrane Binding Studies of Peptides

**Figure 8:**
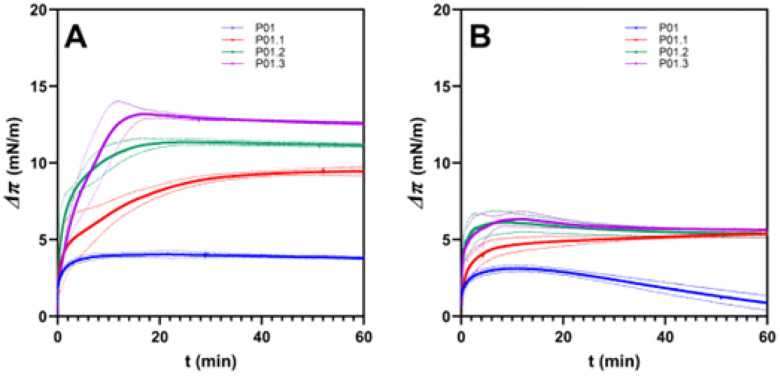
Delta surface pressure (Δπ) of peptides in (A) 7:3 POPG-POPE as Gram-positive bacterial inner membrane model, and in (B) 3:7 as Gram-negative bacterial inner membrane model. The dashed line is the standard deviation of delta surface pressure for each measurement.

The antibacterial activity of these peptides was proposed to be due to their ability to bind and disrupt the integrity of bacterial membranes. Therefore, a Langmuir trough technique, combined with measurement of surface tension was used to monitor the adsorption of the different peptide derivatives to two different membrane models: a mixture of 7:3 POPG/POPE and 3:7 POPG/POPE to mimic Gram-positive and Gram-negative bacteria membranes, respectively. Each peptide was injected into the trough at a concentration of 1.0 μg/mL and the adsorption of the peptides to the membrane covered (Fig. 8) or blank interface (**Figure S1**) are recorded. Figure 8 shows the surface pressure difference (Δπ) before and after peptide injection below the model lipid membranes, as a function of time [24].

In the model of Gram-positive bacterial membrane, P01 peptide absorbed quickly to the membrane and reached a constant surface pressure after 8 minutes of observation with a final delta surface pressure (Δπ) of 4 mN/m (Fig. 8A). In contrast, P01.1 peptide showed a sharp initial increase of 5 mN/m, followed by a more gradual change in surface pressure, reaching a final Δπ value of 9.45 mN/m after 50 minutes of absorption. P01.2 peptide reached a constant surface pressure after 25 minutes with a delta final surface pressure of 11.15 mN/m. The best absorption was shown by P01.3 peptide with a final delta surface pressure of 12.6 mN/m after 27 minutes of observation. A higher value of Δπ indicated that the peptide exhibited greater binding affinity to the lipid monolayer either through hydrophobic or electrostatic interactions [25]. The fact that P01.1 peptide with a higher total charge (+ 6) induced a lower Δπ than P01.3 peptide (+ 4) suggests that the total charge does not result in a greater membrane activity. Several other factors have been suggested to influence the membrane modulatory activity of peptides, including oligomer formation, helicity, amphipathicity, and conformational stability [26]. Our results suggest that helix structure stability and amphipathicity influenced the absorption of peptides to the 7:3 POPG/POPE membrane. In summary, our adsorption data shows that the activity of the derivatives follows as: P01.3 > P01.2 > P01.1 > P01.

In the Gram-negative membrane model, with less POPG and therefore a lower negative charge for the membrane, the three peptides (i.e., P01.1, P01.2, and P01.3) exhibited similar Δπ maximum at 60 min time point while P01 peptide has lower Δπ maximum (Fig. 8B). P01 peptide reached a maximum at 10.32 min with a maximum surface pressure of 3.11 mN/m. After reaching the maximum Δπ, P01 peptide decreased the delta surface pressure gradually; the decrease in surface pressure was presumably due to weakening of the interaction between the peptide and the membrane’s headgroup or due to instability in the membrane induced by disruptive peptide-membrane interactions. The P01.2 and P01.3 peptides showed a rapid increase in Δπ and stabilized at values of 5.61 mN/m after 20 min; in contrast, P01.1 exhibited a gradual increase in Δπ to reach a maximum Δπ of 5.40 mN/m at 60 min time point [27]. Compared to the adsorption to the Gram-positive bacteria, the net change in surface pressure is lower for the Gram-negative bacteria with a lower % of POPG lipid headgroups, suggesting that electrostatics does play some role. However, the differences in the helicity and/or amphipathicity of the peptides do not influence their activity to the Gram-negative bacterial membrane.

### Antibacterial Activity of Peptides

Antibacterial activity of peptides was determined in growth inhibition assay against Gram-negative bacteria *Escherichia coli* (ATCC 23522) and Gram-positive bacteria *Staphylococcus aureus* (ATCC 29312). The native peptide (P01) with a disordered secondary structure was inactive against both bacteria at 0 to 250 μg/mL concentrations (Table 4). In contrast, all three derivative peptides have identical activity against *E. coli* with MIC of 3.91 μg/mL. In the *S. aureus*, P01.3 has the highest activity with a MIC of 15.63 μg/mL, which is four-fold lower activity than a positive control Streptomycin (3.91 μg/mL). The P01.2 peptide inhibited *S. aureus* lower activity (125 μg/mL) than P01.3 while P01.1 has the lowest active of the three derivatives (> 250 μg/mL). It should be noted that the rank of helical structure stability of these derivatives is as follows P01.3 > P01.2 > P01.1 > P01. In summary, there is a correlation between the helicity content in the peptide and its activity against *S. aureus*. Compared to P01 peptide, the helicity of P01.1, P01.2, and P01.3 correlated with their increased activity against *E. coli*.

### Correlation of Structure, Membrane Binding, and Antibacterial Activity

AMR becomes a new challenge in the 21st century. Previously, several small molecules such as β-lactam, aminoglycoside, and quinolones were used and developed to combat microbial infection. These small molecules commonly target metabolic pathways such as protein synthesis, cell wall synthesis, and nucleic acid synthesis to inhibit bacterial growth [28]. The antibiotic resistance mechanisms of microbials include the suppression of drug uptake or efflux, modification of drug target, and drug inactivation [2]. To combat AMR, alternative strategies are being investigated including the membrane disruption mechanism that has been shown to minimize the antimicrobial resistance [29].

AMPs are a class of compounds that cause bacterial membrane disruption to generate membrane pores causing cell leakage to the bacteria [30]. AMPs also have intracellular and extracellular mechanisms to target receptors to cause a metabolic dysfunction [31]. The unique characteristics of AMPs as membrane disrupter include an amphipathic α-helical structure as well as a high total of positive charges [32]. AMPs serve as a defense mechanism for organisms as a part of their immune response against invaders [33].

Several approaches have been used to discover AMPs from an organism, including proteomic method, protein enzyme digestion, and bioinformatic sequencing [34–36]. P01 peptide was obtained from an active fraction of macroalgae *Chondrus crispus* hydrolysates and it has high hydrophobicity and low amphipathic structure (Table 1). Modifications of P01 peptide were carried out via capping C-and N-termini and amino acid mutations to increase helical propensity and amphiphilicity in P01.1, P01.2, and P01.3 peptides (Table 1, Fig. 1). Capping both termini could enhance the plasma stability by preventing exopeptidases degradation and terminus capping has been shown to improve antibacterial activity against Gram-positive multidrug resistance (MDR) bacteria [37]. P01.1 peptide with amidated C-terminus enhanced the helical propensity compared to the parent P01 peptide; furthermore, P01.2 peptide with both termini amidated and acetylated increased the helical structure (*f*_H_) and helical stability at low pH compared to P01 and P01.1 (Figs. 3–4). Further mutation of P01.2 peptide to make P01.3 peptide generated structural amphiphilicity with increased the helical stability. The rank of helical stability was as follows P01.3 > P01.2 > P01.1 > P01 peptides.

It has been previously shown that the degree of separation between hydrophobic and hydrophilic residues in the helical structure has effect on peptide membrane binding [38]. In this study, the selectivity and bioactivity of the peptides (P01, P01.1, P01.2, and P01.3) in Gram-positive *S. aureus* bacteria correlated very well with the increase in amphipathicity and helicity structures (Table 4).[6] In addition, the antimicrobial activity also correlated very well with the observed change in the surface pressure in the model Gram-positive lipid membranes Δπ, induced by the peptides (i.e., P01.3 > P01.2 > P01.1 > P01). In contrast, Δπ values of peptide derivatives (P01.1, P01.2, and P01.3) adsorbing in a model of Gram-negative membranes were approximately the same and their activities were also the same for *E. coli*, a Gram negative bacteria (Table 4; Fig. 8B); however, in the absence in a helical structure and a low Δπ value of P01 peptide, it was inactive against in *E. coli*. Although some antimicrobial peptides are unstructured in water, their conformation can change into a helical structure while interacting with the bacteria membranes [39]. Substitutions of helix breaker residues in P01 peptide (i.e., Pro, Thr, Val) with helix forming residues (i.e., Lys, Glu) in peptide derivatives (i.e., P01.1, P01.2. and P01.3) resulted in the increase helicity. Furthermore, strategic mutation of Thr5 to Glu5 in P01.3 peptide generated a potential salt bridge between the Glu5 residue with Lys2 or Lys8 within the hydrophilic face (Table 1; Fig. 1; Fig. 8D).

The P01.3 peptide has the highest helical content compared to other analogs (i.e., P01, P01.1, P01.2) and the NMR and MD simulation data confirmed the presence a continuous helical structure from Glu5 to the C-terminal; the potential helical break was found Ile3-Val4 (Fig. 7). In this case, the Lys1-Lys2-Ile3-Val4 involved β-turn conformation. Although there was a break at Ile3-Val4, Ile and Val residues have been shown to stabilize the helical structure in membranes because their side chains formed hydrophobic interactions with the lipid chain of the membrane. The LKKL motif in the P01.1, P01.2, and P01.3 peptides was found in some α-helical AMPs with biological activity in Gram-positive and Gram-negative bacteria [40].

The proposed mechanism of action of these peptides in killing both *E. coli* and *S. aureus* bacteria is due to the disruption of the cell membranes to cause bacterial cell leakage. The cell membrane leakage is due to insertion or incorporation of the peptide as monomer and/or oligomers to form membrane pores or weaken the membrane integrity [41]. In general, the membrane binding properties of peptide analogs (i.e., P01.1, P01.2, P01.3) were higher to a model of Gram positive than Gram negative membranes (Fig. 8). Further, while the membrane binding ability correlated with helical stability of peptide, this correlation was more pronounced in Gram positive (Fig. 8A) than in Gram negative (Fig. 8B) membranes, which was also reflected in the peptide’s antibacterial activity. In the model membranes and Langmuir-through measurements, all analog peptides exhibited higher Δπ against the Gram-positive bacterial membrane model with 7:3 POPG/POPE than Gram-negative bacteria with less POPG (Figs. 5A–B). We note that these amphipathic peptides did not demonstrate any surface activity when injected below a blank membrane-free surface, suggesting that the amphiphilicity of the peptide itself was not enough to cause an adsorption, and the change in surface pressure observed is enabled by lipid-protein interactions, possibly due to electrostatic interactions between the lipid and peptides (**Supplementary Figure S1**) [42]. The POPG lipid has more negative charges than POPE, making it favorable for the positively charged peptide to interact with an anionic headgroup such as POPG via electrostatic interaction [43]. However, a higher total positive charge did not directly contribute to a higher membrane affinity of the peptide (Fig. 5A). This suggests that the affinity of peptides to the membrane was not only driven by electrostatic interaction but also by the structure stability and amphipathicity [26].

It was also proposed that dimerization or oligomerization (Fig. 5, Table 2) properties of the peptide could influence their biological activity. It has been proposed that the helical antimicrobial peptide has the ability to self-assemble into an oligomer during its interaction with membranes to create membrane pores [44]. In common a barrel-stave mechanism, peptide-peptide interaction was formed to stabilize a pore structure that caused cytoplasm leaking [45]. The K_D_ dimerization of P01.3 peptide (5.15 ×10^− 5^ M) was lower than P01.1 peptide (7.69 ×10^− 4^ M). The higher dimerization property of P01.3 than P01.1 peptides correlated with the higher membrane binding properties of P01.3 compared to P0.1 peptide. This higher dimerization property of P01.3 peptide also reflected in its antibacterial activity in Gram positive *S. aureus* compared to P01.1 peptide. However, the level of contribution of dimerization compared to charge, helicity, and amphiphaticity of the peptide was difficult to separate. In the future, the oligomerization properties of these peptides in model membranes will be evaluated.

## Conclusion

In this study, three strategies (i.e., terminus capping, residue mutation, and potential salt bridge formation) were implemented to parent P01 peptide to improve the helical structure stability and amphiphilicity. These modifications provided P01.3 peptide with the highest helical stability with the best antibacterial activity in *E. coli* and *S. aureus*; however, the correlation between helicity and biological activity was more pronounce in *S. aureus* than in *E. coli*. The mechanism of biological activity of these peptides was due to their membrane interaction properties to create leakiness in the bacterial membranes.

## Materials and Methods

### Design of analog antimicrobial peptide

Antimicrobial peptide P01 (KKNVTTLAPLVF), as the model peptide, was obtained from our previous studies [8]. Several amino acids were substituted to optimize the peptide physicochemical properties. The first strategies to optimize the physicochemical properties of peptides were accomplished by increasing hydrophobicity, increasing total charge, building the amphipathic structure, and capping of N- and C-termini. Hydrophobicity enhancement was done by substituting Asn3 and Thr6 residues with Ile3 and Ile6 residues. The total charge and amphipathic structure were increased by substituting Ala8, Pro9, and Phe12 residues with lysine. The C-terminal of the peptide was amidated, the product of the first strategy was the P01.1 peptide (KKIVTILKKLVK-NH_2_). The second strategy to enhance peptide activity was N-terminal acetylation, producing P01.2 peptide (Ac-KKIVTILKKLVK-NH_2_). Capping both of N- and C-termini is a common strategy to increase the peptide stability against exopeptidase enzymes. The last strategy to enhance the activity of the peptide was done by substituting Thr5 with Glu5 as a high helical propensity residue to make P01.3 peptide (Ac-KKIVEILKKLVK-NH_2_). All peptides were synthesized by DG peptide (Shanghai, China) with 95% purity.

The physicochemical properties of each peptide (i.e., charge and molecular weight) were analyzed using ProtParam from Expasy (https://web.expasy.org/protparam/). The Heliquest web server (https://heliquest.ipmc.cnrs.fr/index.html) was used to analyze the hydrophobicity (H) and mean vector of the hydrophobic moment (μH) of each peptide. The toxicity was predicted by ToxinPred (https://webs.iiitd.edu.in/raghava/toxinpred/).

### Circular dichroism spectroscopy

The secondary structure and structural stability analyses were conducted by circular dichroism spectroscopy. All spectra were recorded by Jasco-815 spectrophotometer using a 0.1 cm path length cell. Concentration optimization was done by analyzing 125, 250, 500; and 1000 ppm of peptide P01 in 10 mM sodium phosphate buffer. The secondary structure of the peptides was analyzed in the presence of 0; 5; 10; 15; 20; 30 and 40% TFE in 10 mM sodium phosphate buffer. All CD spectra were converted from millidegrees (m°) to molar relative ellipticity ([θ]) using Eq. 1:

Eq. 1 $$[\theta ]=\frac{{\text{m}}^{\circ }.\text{M}}{10.L.C}$$

with M as the average molecular weight (g/mol), C as concentration (g/L), and L as the path length of the cell (cm). All analysis was carried out in 5 scans with wavelength region of 195–250 nm. All the mean helicity value (*f*_H_) were calculated using Eq. 2:

Eq. 2 $$f_{H}=\frac{{\left[\theta \right]}_{222}-(1550-40T)}{\left(-42,400+140T)\left(1-\frac{4.8}{N_{pep}}\right)-(1550-40T\right)}$$

where T and N_pep_ are for temperature assay and number of peptide units (N_res_ + 1), respectively [46].

Peptide stability assays were conducted at different temperatures and pHs. Temperature variations used in the study were between 10–8 °C with a temperature increment of 5 °C /point. All peptides were diluted to 0.1 M phosphate-citrate buffer at pH 3–8. All stability evaluations were carried out in 20% TFE solution as a starting point for the helical formation. All analysis was carried out in 5 scans at the wavelength region of 190–240 nm.

Concentration-dependent CD spectroscopy was carried out to evaluate the dimer formation of each helical peptide. Signal intensities at 222 nm with different concentrations were collected. Peptide CD spectra were determined with concentrations of 1.0 ×10^−3^ − 2.5 ×10^−6^ M for P01.3 and 1.0 ×10^−3^ − 2.5 × 10^− 5^ M for P01.1. All peptides were diluted in 40% TFE in water (v/v). Concentration-dependent dimerization was analyzed with a nonlinear curve fitting method by fitting concentration and observed molar relative ellipticity ([*θ*]_*obs*_) to Eq. 3,

Eq. 3 $${\left[\theta \right]}_{obs}={\left[\theta \right]}_{d}+\left({\left[\theta \right]}_{m}-{\left[\theta \right]}_{d}\right)\frac{-K_{D}+\sqrt{K_{D}^{2}+4C_{t}K_{D}}}{2C_{t}}$$

where *C*_*t*_ is the total concentration. The molar relative ellipticity of dimer ([*θ*]_*d*_) and monomer ([*θ*]_*m*_), and the dissociationconstant of monomer and dimer (K_D_) were determined parameters in Eq. 3 [16].

### Nuclear magnetic resonance and molecular dynamics simulation

A three-dimensional (3D) peptide structure was generated using 2D-NMR spectroscopy and molecular dynamic (MD) simulation [47]. 2D-NMR analysis was carried out by Bruker Avance 600 MHz at 25 °C. The peptide was diluted by 30% MeOD-d_4_ in water (v/v) with 3000 ppm concentration. The dihedral angle (*Φ*) of the peptide was determined by the Karplus equation (Eq. 4) using the ^3^J_NHHα_ coupling constant with *θ* = |60 − *Φ*| [48]. Interproton distance (R_0_) was calculated by Eq. 5 using the Nuclear Overhauser Effect (NOE) intensity (I_0_). The geminal protons distance (R_S_ = 1.7 Å) and NOE intensity (I_S_) were used as a standard for distance calculation of two protons.

Eq. 4 J3NHHα=6.4Cos2θ−1.4Cosθ+1.9

Eq. 5 $$\frac{I_{o}}{I_{s}}=\frac{R_{s}^{-6}}{R_{o}^{-6}}$$

The three-dimensional structure of the peptide was calculated by NOE distance restraint and coupling constant using the ARIAweb server [23]. The generated structure from ARIA was analyzed by the PROCHECK on the SAVES v6.1 webserver (https://saves.mbi.ucla.edu/) [49]. The best-generated structure was used as the initial structure for NMR structure refinement by Gromacs v5.1.4. MD system was generated using solvation builder CHARMM-GUI with 0.15 M NaCl in a cubic water box of size 4.468 nm [50]. The system consisted of 9007 total atoms, with 242 protein atoms, 8 Na + and 12 Cl- ions, and 2915 TIP3P model waters [51]. The CHARMM36m forcefield was used in the simulation [52]. A brief energy minization was performed with the steepest descent algorithm [53]. A 10 ns equilibration was done using a V-rescale thermostat for 10 ns under isothermal-isobaric (NpT) conditions. The temperature was maintained at 300 K with a time constant of 0.5 ps. The pressure was maintained at 1 bar using isotropic pressure coupling with Parrinello-Rahman barostat with a 1 ps time constant and compressibility of 4.56 × 10 − 5 (kJ·mol^− 1^·nm^− 3^)^−1^ [54]. The production run was performed using CHARMM36m force field for 100 ns simulation under NVT conditions at 300 K [55]. The results analysis and visualization were performed by VMD and BIOVIA Discovery Studio [56].

### Membrane studies

Peptide adsorption to model lipid membranes were carried out to determine the absorption ability of peptides to the inner bacterial membrane model. Two membrane models were made from palmitoyl-oleoyl-phosphatidyl-ethanolamine (POPE) and palmitoyl-oleoyl-phosphatidyl-glycerol (POPG). Gram-negative bacteria were represented by 3:7 P0PG/POPE, whereas Gram-positive bacteria inner membranes were represented by 7:3 POPG/POPE [57]. The membrane was created by spreading the respective POPG/POPE solutions in chloroform on the water surface until a surface pressure of around ~ 30 mN/m was reached, to mimic the membrane equivalent surface pressure of inner bacterial membranes [58]. The chloroform was allowed to evaporate and the membrane was allowed to equilibrate by waiting for 20–30 minutes after spreading. To record the absorption of the peptides to the monolayer membrane, 1 μg/mL of peptide was injected into the bulk solution, using a hole drilled into the side of our trough. The absorption was evaluated by recording the surface pressure difference (Δπ) as a function of time after peptide injection into the membrane, using a filter paper Wilhelmy plate set-up. The surface pressure of the system was evaluated for 1 h with peptide injection point as starting time (t = 0).

### Antibacterial activity assay

Antibacterial assay was performed by microbroth dilution method against *Escherichia coli* ATCC 23522 and *Staphylococcus aureus* ATCC 29312 to obtain minimum inhibitory concentration (MIC). The bacteria inoculant was prepared by growing the bacteria in the Mueller-Hinton broth (MHB) for 24 h and diluted until ~ 10^5^ CFU/mL for the assay. Streptomycin and sterile water were used as control positive and control negative. The peptide/Streptomycin stocks of 500 μg/mL were prepared by dissolving solid peptides in sterile water and diluted to 0.49–250 μg/mL in the sterile polystyrene 96-well plate. About 10 μL bacteria inoculants and 40 μL MHB media were transferred into the well plate and incubated at 37 °C for 24 h. The bacterial growth was analyzed by a microplate reader at 650 nm wavelength. The peptide MICs were calculated by comparing inoculant-treated peptide absorbance with control negative absorbance.

## Supplementary Files

This is a list of supplementary files associated with this preprint. Click to download.

- GA.png
- SupplementaryMaterial.docx

## Figures and Tables

**Table 1:** Physicochemical properties of P01 and its derivatives

Peptide	Sequence	Charge	MW^[a]^ (Da)	<H>^[b]^	<μH>^[b]^	Toxicity^[c]^
P01	**KKNVTTLAPLVF**	+ 2	1330.63	0.550	0.246	Non-toxic
P01.1	**KKIVTILKKLVK-NH**_**2**_	+ 6	1409.89	0.396	0.746	Non-toxic
P01.2	**Ac-KKIVTILKKLVK-NH**_**2**_	+ 5	1451.89	0.396	0.746	Non-toxic
P01.3	**Ac-KKIVEILKKLVK-NH**_**2**_	+ 4	1479.90	0.321	0.821	Non-toxic

[a] MW = molecular weight in Dalton (Da)

[b] < H > = Hydrophobicity and < μH > = mean vector hydrophobicity determined by Heliquest web server

[c] Toxicity was predicted by ToxinPred web server

**Table 2:** The dimerization dissociation constant (KD) of peptides

Peptide	K_D_ (M)^[a]^	[θ]_monomer_	[θ]_dimer_	COD^[b]^
P01.1	7.69 ×10^−4^	−220000	−100000	0.92
P01.3	5.15 ×10^−5^	−760000	−75000	0.99

[a] Determined by nonlinear least-square fitting method with Eq. 3.

[b] COD = Coefficient of determination of fitting curve (Fig. 5).

**Table 3:** 1H-NMR chemical shift of P01.3 peptide determined by COSY, TOCSY and NOESY.

Residue	NH	Hα	Hβ	Hγ	Hδ	^3^J_NH−HCα_ (Hz)	Calculated Phi^a^	Observed Phi^b^	Observed Psi^c^
Acetyl		0.84/0.84							
K_1_	8.26	4.2	1.74	1.43	2.97	6.56	−83.24	155.43	177.81
K_2_	8.37	4.28	1.79	1.42	2.95	7.12	−79.61	−71.58	142.81
I_3_	8.08	4.11	1.88	1.49/1.23	0.89	7.40	−86.60	−50.79	−44.09
V_4_	8.03	3.98	2.05	0.93		7.46	−87.04	−62.95	−44.83
E_5_	8.17	4.28	1.53/1.70	2.38/2.01		6.88	−82.83	−65.68	−42.04
I_6_	8.06	4.03	1.88	1.53/1.19	0.89	8.09	−89.25	−57.01	−41.41
L_7_	8.19	4.24	1.7	1.53	0.89	6.97	−81.37	−57.64	−46.71
K_8_	8.10	4.2	1.79	1.4	2.95	7.38	−81.94	−60.88	−49.29
K_9_	7.99	4.24	1.79	1.40	2.99	7.51	−85.49	−54.49	−43.57
L_10_	8.11	4.33	1.67	1.58	0.89	7.38	−81.94	−69.25	−52.39
V_11_	7.94	4.11	2.09	0.93/0.93		8.67	−97.65	−64.34	−30.139
K_12_	8.22	4.28	1.83/1.75	1.45/1.3	2.97	7.67	−83.24	−55.26	177.93
C-term-NH_2_	7.56/7.10								

a Calculated Phi dihedral angles from ^3^J_NH−HCα_ of each amino acid

b Observed Phi dihedral angles from a representative peptide structure from MD simulations

c Observed Psi dihedral angles from a representative peptide structure from MD simulations

**Table 4:** Antibacterial activity of AMPs against Escherichia coli (ATCC 23522) and Staphylococcus aureus (ATCC 29312).

Peptide	Minimum Inhibitory Concentration (MIC, μg/mL)	
	*Escherichia coli* (ATCC 23522)	*Staphylococcus aureus* (ATCC 29312)
P01	n/a^[a]^	n/a^[a]^
P01.1	3.91	>250.00
P01.2	3.91	125.00
P01.3	3.91	15.63
Streptomycin	<0.49	3.91

[a] inactive in 0.49–250 μg/mL concentrations range
